# Nest Site Selection and Nesting Behavior of Reeves' Turtle (*Mauremys reevesii*) in Qichun County, Hubei Province, China

**DOI:** 10.1002/ece3.71630

**Published:** 2025-06-30

**Authors:** Zihao Ye, Rongping Bu, Hai‐Tao Shi

**Affiliations:** ^1^ Ministry of Education Key Laboratory for Ecology of Tropical Islands, College of Life Sciences Hainan Normal University Haikou China; ^2^ College of Marine Science, Guangxi Key Laboratory of Beibu Gulf Biodiversity Conservation Beibu Gulf University Qinzhou Guangxi China

**Keywords:** clutch size, nest site selection, nesting behavior, threat factors, turtle conservation

## Abstract

Research on nest site selection and nesting behavior is essential for species conservation. Research on the reproductive ecology of Reeves' turtles is relatively scarce. Therefore, from April to September 2022, we conducted a study in Qichun County, China, on the field nest site selection of 11 female Reeves' turtles using tracking line boxes. We also investigated the nesting behavior of 20 female turtles using high‐definition surveillance cameras placed within an enclosure and recorded the clutch size. The results showed that slope, canopy cover, and distance from the water source significantly influenced nest site selection. The nesting behavior was divided into five stages: landing, excavation, egg laying, covering, and return, with a nesting season from early May to the end of July and nesting most occurring during dusk and dawn. The average clutch size was 7.33 ± 0.33 eggs. Reproduction was affected by high nest predation rates and extreme weather conditions. This study provides valuable information on this endangered species and a foundation for the development of conservation plans.

## Graphical abstract

This study investigates the nest site selection and nesting behavior of Reeves' turtles ( *Mauremys reevesii* ) in Qichun County, China. Our findings indicate that slope, canopy cover, and distance from the water source significantly influence nest site selection. The nesting behavior was categorized into five stages: landing, excavation, egg laying, covering, and return. Reproduction was notably impacted by high predation rates and extreme weather conditions.

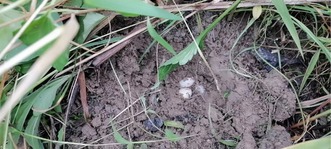

## 1 Introduction

Breeding is a crucial step in turtle population growth (Kuchling 1999). Turtles typically exhibit a low reproductive capacity and lack parental care (Congdon et al. 1993; Warner et al. 2016), instead relying on their extended reproductive lives, longevity, and delayed maturity to provide reproductive opportunities (Gibbons 1987; Congdon et al. 1993). In addition, turtles tend to exhibit increased reproductive capacity with age and high survival rates from age one year to maturity, contributing to population growth under favorable conditions (Segura et al. 2021; Congdon et al. 2022).

Historically, the reproductive strategies of turtles have been well‐adapted to natural environments. However, under the current rapid environmental changes, turtles are more prone to extinction than many other reptiles, amphibians, mammals, and birds. (Stanford et al. 2020; Rhodin et al. 2018). For example, climate change (Pike 2014; Monsinjon et al. 2019), habitat fragmentation (Ennen et al. 2010), and loss of nesting sites (Lazure and Galois 2019) lead to reproductive failure. Additionally, human factors, such as the targeted capture of sexually mature individuals (Sung et al. 2013) and female mortality owing to road crossings during the nesting season (Santori et al. 2018), result in the loss of reproductive individuals. Conducting reproductive ecology research on turtles can provide managers with data that can be used to develop protective measures to better safeguard this group (Roosenburg et al. 2014; Páez et al. 2015), making it a focus of conservation efforts (Mui et al. 2016; Buckardt et al. 2020; Sullivan et al. 2022).

Reeves' turtles ( *Mauremys reevesii* ) are widely distributed in East Asia, including China, Korea, and Japan (Lovich et al. 2011). In China, Reeves' turtles are one of the most widely distributed turtle species (Sowerby 1925; Pope 1935). However, habitat degradation in China, coupled with collection for food, the pet trade, and medicine, has led to their disappearance from half of their historical range (van Dijk 2011). Owing to the threats they face, the Reeves' turtle has been listed as an endangered species since 2011 (IUCN 2022). In Qichun County, Hubei Province, the Reeves' turtle is under severe threat from hunting and habitat destruction (Bu, Ye, Xiao, and Shi 2022). While researchers have studied the habitat selection, activity rhythms, home range, and hibernation of Reeves' turtles in Qichun (Bu, Ye, and Shi 2022; Bu et al. 2023, 2024), research on their reproductive ecology remains relatively scarce.

Existing research on Reeves' turtles has mainly been conducted in laboratories and focused on reproductive physiology (Saka et al. 2011; Jung et al. 2016), hatching (Du et al. 2006), courtship and mating behavior (Koo et al. 2015), and greenhouse cultivation (Sandi et al. 2020). In contrast, fewer studies have been conducted on populations in captive or wild states but include studies on breeding in enclosures (Fukada and Ishihara 1974) and the migration of wild turtles during the breeding season using radio telemetry (Haramura et al. 2010).

Therefore, considering these gaps in the research, this study aims to provide field reproductive ecological data for Reeves' turtles. Specifically, this study primarily encompasses the following three aspects.

### 1.1 Nest Site Selection

The study identifies the nesting sites of Reeves' turtles in the wild and document the ecological factors influencing their selection. These nesting sites are compared with randomly selected locations using statistical analyses to determine the key ecological factors affecting nest site choice. Understanding Reeves' turtle nesting preferences can inform conservation strategies by guiding the protection of critical nesting sites and the assessment of potential habitats for the establishment of protected areas.

### 1.2 Nest Characteristics, Clutch Size, and Egg Characteristics

This study documents fundamental reproductive data on wild Reeves' turtles, including the nest characteristics, clutch size, and egg traits, which remain largely unexplored in natural habitats. The findings will provide valuable insights into reproductive patterns of the species and the potential reproductive challenges.

### 1.3 Nesting Behavior

This study determines the nesting time, temperature, seasonality, and behavioral patterns of this species within its native distribution range. This study was conducted in a semi‐natural enclosure and surveillance cameras were used to observe and record the nesting behavior of egg‐laying female turtles. The findings will serve as a valuable reference for improving conservation efforts for the species.

In summary, the results of this study will provide crucial information to support the development of effective conservation measures for Reeves' turtles and other declining freshwater turtles.

## 2 Materials and Methods

### 2.1 Nest Site Selection

This study was conducted in Qichun County, Hubei Province, China (29°59′–30°40′ N, 115°12′–115°56′ E), from April to August 2022. We captured 11 gravid female wild Reeves' turtles (using a portable X‐ray machine to scan the turtles' lower abdomen to confirm the presence of hard‐shelled eggs (Lin et al. 2018)) (Table 1) using cage traps (Bu, Ye, Xiao, and Shi 2022), which were dismantled after capture to avoid harm to local wildlife. Then, a tracking line box was attached to the rear of the turtle's carapace. The boxes were designed based on schemes developed by Li (2013) and Yang (2014), the tracking line box was affixed to the third costal scute using a 1:1 mixture of epoxy resin and epoxy resin curing agent as the adhesive. A fishing line was wound around the pulley to a length of approximately 70 m. The total weight of the tracking line boxes and adhesive did not exceed 40 g (10% of the turtle's body weight) and thus did not affect the turtles' normal movement (Beaupre et al. 2004). The Reeves' turtles were then released where they were captured. To prevent the fishing line from running out and affecting the turtle's normal activity, the specific locations of the turtles were determined at 06:00, 14:00, and 20:00, and the fishing line was reeled in. Signs of nest construction were checked along their path to identify the nests (Yang 2014; Lin et al. 2018). After the study was completed, all tracking line boxes were carefully removed and the turtles were released into the aquatic habitats nearest to their nesting sites.

**TABLE 1:** Ecological survey tracking of 11 gravid female Reeves' turtles in the wild in Qichun County, Hubei Province, China.

Number	Carapace length (mm)	Carapace width (mm)	Plastron length (mm)	Plastron width (mm)	Weight (g)
1	177.10	112.94	167.16	113.00	897.4
2	143.34	95.10	140.88	83.90	471.6
3	198.60	120.40	182.60	103.50	1034.0
4	136.08	93.10	126.38	81.20	406.9
5	146.22	97.32	136.68	97.00	467.3
6	145.01	97.28	127.66	81.00	446.1
7	163.52	102.66	152.68	87.00	710.3
8	185.90	126.90	171.36	108.34	978.4
9	170.90	113.50	163.32	113.20	799.8
10	140.68	91.56	130.00	75.50	404.8
11	182.18	119.40	179.40	106.70	845.7

Once a nest site was located, two quadrats centered on the nest were established. In the small quadrat (1 × 1 m), soil hardness, leaf litter thickness, shrub and herb cover, and height were measured. In the large quadrat (5 × 5 m), the slope, slope position (flat ground = 1, lower slope position = 2, mid‐slope position = 3, and upper slope position = 4), slope direction (northeast direction = 1, southeast direction = 2, southwest direction = 3, and northwest direction = 4), distance from human disturbance, canopy cover, vegetation type (grassland = 1, shrub‐grassland = 2, and tree‐shrub woodland = 3), distance from the water source, and height above the water source were measured. For each selected quadrat, a random quadrat was established within 10–50 m in a random direction, positioned using a random function in Microsoft Excel. The minimum separation of 10 m ensured no overlap between the random and selected quadrats, while the maximum separation of 50 m was based on the average home range length of Reeves' turtle (Song et al. 2014). The same ecological factors were measured for the random quadrats.

### 2.2 Nest Characteristics, Clutch Size, and Egg Characteristics

After locating the nest, the eggs were gently excavated while minimizing damage to the nest structure. The side of the egg with the fertilization spot facing upward was placed and the nest depth, inner width, entrance diameter, egg length, and egg width were measured using Vernier calipers. Egg weight was measured using an electronic balance and the number of eggs in the clutch was recorded. After measurements were taken, the eggs were placed back into the nest and the nest was restored to its original condition.

### 2.3 Nesting Behavior

At a semi‐natural pond (artificially excavated for agricultural irrigation and later abandoned) within the study site, an irregularly shaped enclosure measuring approximately 5 m in length and 3 m in width (total area ~15 m^2^) was constructed using iron railings and wire mesh. The enclosure's dimensions were selected to optimize operational efficiency for investigating nesting behavior, with the smaller size allowing comprehensive coverage using two surveillance cameras (Hikvision, DS‐2CD2225CD‐LGLSE, Hangzhou, China). The enclosure had a ratio of aquatic to terrestrial areas of approximately 2:3 and was equipped with fallen logs, floating logs, and emergent plants (eg: Cattail (*Typha* spp.); Reed ( *Phragmites australis* )), floating plants (e.g., Water Caltrop (*Trapa bispinosa Roxb*.)). In addition, a variety of herbaceous (including abundant naturally growing herbaceous plants with unidentifiable species), climbing (eg: Japanese Honeysuckle ( *Lonicera japonica* ); Kudzu ( *Pueraria montana var. lobata* )), and woody plants (eg: Camphor Tree ( *Cinnamomum camphora* ); Oil‐tea Camellia (*Camellia oleifera*)) were present on the shore. Two surveillance cameras were placed on opposite sides of the enclosure to ensure full monitoring coverage and a temperature logger was positioned on the soil surface to record the nearby temperature every hour.

To facilitate capturing sufficient behavioral data, 20 sexually mature wild female Reeves' turtles were selected. Considering that some individuals' eggs may not have yet developed into the hard‐shelled stage, relying solely on portable X‐ray screening to confirm the presence of hard‐shelled eggs may result in the exclusion of potential study subjects. Therefore, any individual that met the criteria for sexual maturity was included in the study. The turtles were captured using cage traps, marked with white waterproof tape on their carapaces for individual identification, and introduced into the enclosure. The monitoring system was operational 24 h a day from May 25th to September 1st, covering the entire nesting season (Fukada and Ishihara 1974; Saka et al. 2011). During the study, videos from the monitoring system were downloaded and watched daily to observe nesting behavior. Data such as the time of occurrence, total duration, duration of each behavioral phase, and weather were recorded. After completion of the study, all turtles were released into several aquatic habitats where we had previously captured individuals of this species.

To facilitate behavioral observation and quantification, we defined distinct phases in the nesting behavior of Reeves' turtle as follows: Landing (LD): Begins when a female emerges from water and concludes prior to nest excavation initiation, encompassing the search for suitable nesting sites along the shoreline. Excavation (EX): Begins with the initiation of digging motions and terminates upon cessation of digging immediately preceding the deposition of the first egg, representing the nest cavity formation process. Egg Laying (EL): Extends from immediately before the first egg deposition to completion of the final egg deposition, encompassing the entire oviposition sequence. Covering (CO): Begins with hind limb movements to refill the nest cavity with substrate and concludes when the female abandons the nest site, corresponding to the complete burial process. Return (RE): Begins with the female's retreat from the nest site and ends with her return to the aquatic environment.

### 2.4 Statistical Analysis

All statistical analyses were conducted using SPSS 26.0 (SPSS Inc., Chicago, IL, USA). In the field tracking studies, 12 ecological variables were collected to identify habitat preferences of Reeves' turtles during nest site selection. Comparative analyses were performed between selected nests and random quadrats. To achieve the objectives, we employed three analytical methods: (1) conventional difference tests: The normality of the data was assessed using the Kolmogorov–Smirnov test before further analysis. Paired‐sample *t*‐tests were used to analyze significant differences in ecological factors between the selected and random quadrats if the data were normally distributed and had homogeneous variances. Descriptive statistics were reported as mean ± standard error (SE). If the data were not normally distributed, the Wilcoxon signed‐rank test was used and the results are presented as medians (minimum, maximum). For qualitative data, significant differences were determined using the chi‐squared test after calculating the frequency distribution. The significance level was set at *p* < 0.05. (2) Discriminant function analysis (DFA): To identify key ecological factors influencing nest site selection in Reeves' turtles, linear discriminant analysis was conducted. Prior to analysis, variables were preprocessed as follows: continuous variables (e.g., slope, canopy cover) were standardized using *Z*‐scores, while categorical variables (e.g., slope position, vegetation type) were dummy‐coded, with three reference categories manually excluded: slope position = 4, slope direction = 4, and vegetation type = 3. Stepwise discriminant analysis was performed using statistical software, with variables entered into the model based on a significance threshold of *p* < 0.05 (Wilks' Lambda criterion). Equal prior probabilities were assumed for all groups (selected vs. random quadrats). Model performance was evaluated via leave‐one‐out cross‐validation to ensure robust classification accuracy. (3) Principal component analysis (PCA): To identify key ecological factor combinations influencing nest site selection in Reeves' turtles, PCA was applied to reduce the dimensionality of 12 ecological variables. Data preprocessing mirrored the steps used in discriminant function analysis. The suitability of the data for PCA was confirmed by a Kaiser‐Meyer‐Olkin test and Bartlett's test of sphericity. Principal components were extracted based on the Kaiser criterion (eigenvalues > 1) and rotated using Varimax rotation to enhance interpretability. Principal component scores were computed, and independent paired samples *t*‐tests were conducted to assess differences in these scores between selected quadrats and random quadrats. The significance level was set at *p* < 0.05.

Nest characteristics, clutch size, egg characteristics, and nesting behavior were presented as mean ± SE. The significance level was set at *p* < 0.05.

## 3 Results

### 3.1 Nest Site Selection

**FIGURE 1:**
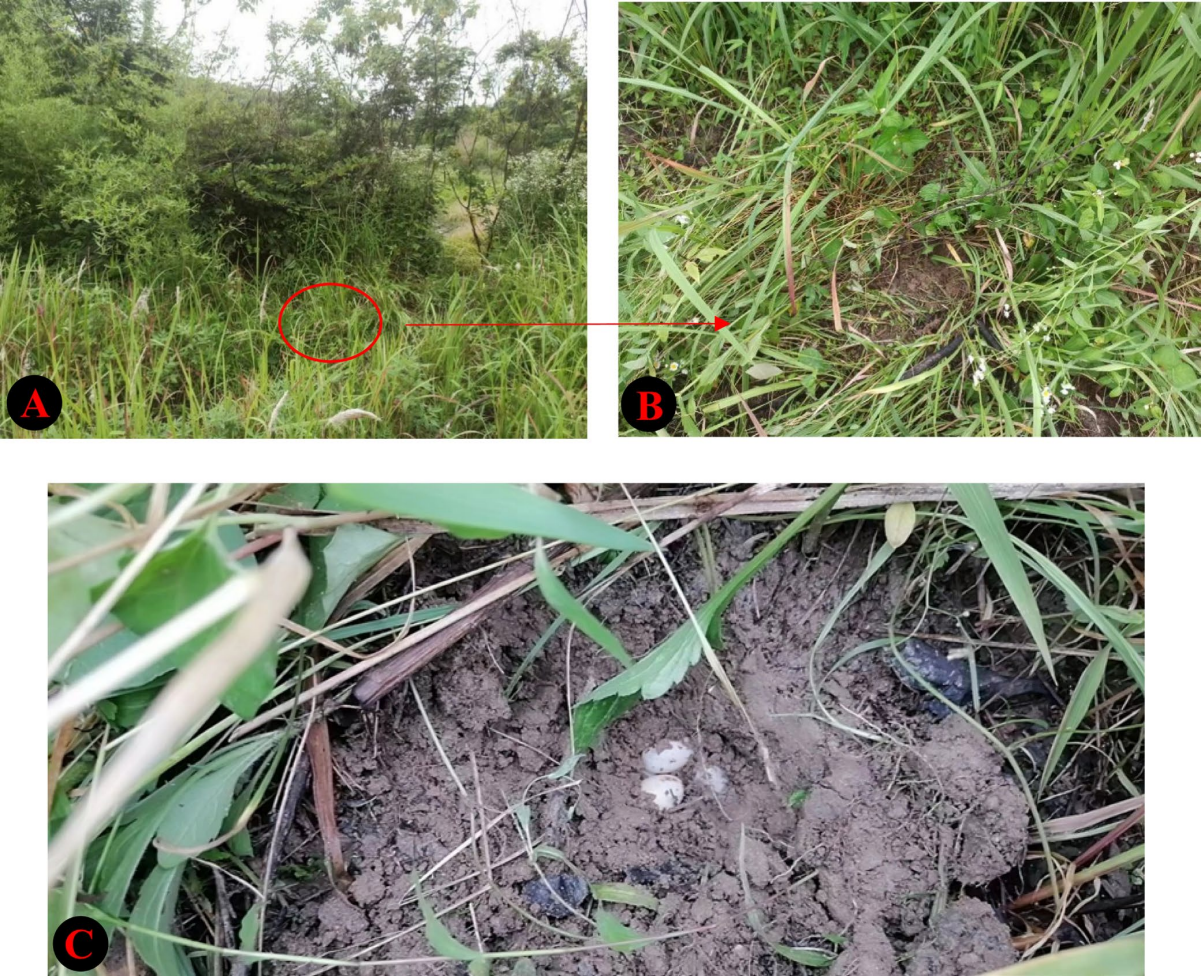
Habitat where a certain turtle selects a nesting site, with the red circle representing the location of the nesting site (A and B). Nest in the wild (C).

For the 11 tracked turtles, seven nests were located, with one of turtles making two nests and no nests found for five of the turtles. In addition, we discovered a nest site from a turtle that was not trapped for this study and included it in the analysis. Therefore, eight nest sites were used for the nest site selection study. Significant differences were observed in the selection of the three ecological factors by turtles in the large quadrats measuring 5 × 5 m. These factors were slope (*t* = −2.883, df = 7, *p* = 0.024), canopy cover (*t* = −2.510, df = 7, *p* = 0.040), and distance from water source (*t* = −2.711, df = 7, *p* = 0.030) (Table 2). However, no significant differences were observed in the small quadrats. In summary, when choosing nesting sites, Reeves' turtles tend to select locations with gentler slopes (4.63° ± 2.34°), lower canopy coverage (28.75% ± 5.73%), and closer proximity to water sources (15.19 ± 7.49 m) (Figure 1A,B).

**TABLE 2:** Differences in 13 ecological factors between selected and random habitats.

Quadrat size (m)	Ecological factor	Mean ± SE or median (minimum, maximum)		Paired *T*‐Test or *χ* ^2^‐test		
		Selected quadrat	Random quadrat	*t* or *χ* ^2^	df	*p*
5 ⋅ 5	Slope (°)	4.63 ± 2.34	15.50 ± 4.18	−2.883	7	0.024
5 ⋅ 5	Slope position	—	—	4.80	3	0.187
5⋅5	Slope direction	—	—	0.476	3	0.924
5 × 5	Distance from human disturbance (m)	20.69 ± 12.49	24.31 ± 11.32	−0.906	7	0.395
5 × 5	Canopy cover (%)	28.75 ± 5.73	58.75 ± 10.03	−2.510	7	0.040
5 × 5	Vegetation type	—	—	2.571	2	0.276
5 × 5	Distance from water source (m)	15.19 ± 7.49	21.75 ± 8.44	−2.711	7	0.030
5 × 5	Height above water source (m)	4.93 ± 2.50	6.50 ± 2.59	−1.052	7	0.328
1 × 1	Soil hardness (Kg/cm^2^)	4.56 ± 0.46	4.17 ± 0.39	0.604	7	0.565
1 × 1	Leaf litter thickness (cm)	1.38 ± 0.32	2.25 ± 0.59	−1.078	7	0.317
1 × 1	Shrub and herb height (cm)	37.88 ± 8.72	67.50 ± 19.69	−1.551	7	0.165
1 × 1	Shrub and herb cover (%)	55.00 ± 12.18	40.62 ± 13.10	1.054	7	0.327

When conducting discriminant analysis between selected quadrats and random quadrats, the eigenvalue of stepwise discrimination was 0.482 and the canonical correlation coefficient was 0.570, accounting for 100% of the variance. Wilks' *λ* indicated a significant difference between the selected and random quadrats (Wilks' *λ* = 0.675, *χ* ^*2*^ = 5.307, df = 1, *p* = 0.021). Stepwise discriminant analysis demonstrated that canopy coverage was a significant distinguishing factor between the selected and random quadrats, with a correct discrimination rate of 75.0% (Table 3).

**TABLE 3:** Stepwise discriminant analysis between selected and random habitats.

Comparison type	Factor	Discriminant Coefficient	Wilks' *λ*	*F*	*p*
Selected and random quadrat	Canopy cover	0.043	0.675	6.742	0.021

During the PCA of the dataset, the Kaiser‐Meyer‐Olkin test yielded a value of 0.73 (> 0.6), indicating the suitability of the data for PCA. Bartlett's test of sphericity confirmed significant correlations among the variables (*χ* ^2^ = 210.5, *p* < 0.001). The total variance explained by the principal components (Table 4) revealed that the first five components had eigenvalues > 1, cumulatively accounting for 83.89% of the total variance. Independent paired samples *t*‐tests on the scores of these five principal components (Table 5) showed that PC1 exhibited a significant between‐group difference (*t* = 4.046, df = 7, *p* = 0.005), with a negative mean difference of −1.129, indicating that random quadrats had significantly higher PC1 scores than selected quadrats. Since no significant differences were detected for PC2–PC5, the high‐loading variables of PC1 were identified as critical factors influencing Reeves' turtle nest site selection.

**TABLE 4:** Total variance explained by principal components.

Factor	Total	Percentage of variance	Cumulative percentage
1	5.183	30.487	30.487
2	3.269	19.231	49.718
3	2.627	15.453	65.171
4	1.801	10.596	75.767
5	1.381	8.123	83.890
6	0.856	5.034	88.924
7	0.647	3.804	92.782
8	0.493	2.899	95.627
9	0.250	1.468	97.095
10	0.220	1.294	98.390
11	0.172	1.011	99.401
12	0.062	0.367	99.768
13	0.026	0.151	99.919
14	0.011	0.062	99.982
15	0.003	0.018	100.000

**TABLE 5:** Results of independent paired samples *t*‐test for the significance of between‐group differences in principal component scores.

Factor	*t*	df	*p*	Mean difference	95% confidence interval
1	−4.046	7	0.005	−1.129	[−1.789, −0.469]
2	−0.767	7	0.468	−0.083	[−0.339, 0.173]
3	−1.179	7	0.277	−0.577	[−1.735, 0.580]
4	−1.523	7	0.172	−0.578	[−1.476, 0.320]
5	−1.571	7	0.160	−0.208	[−0.521, 0.105]

Positive loadings in PC1 suggest that turtles avoid habitats with higher values of these variables and prefer lower values. Varimax rotation (Table 6) further clarified the structure of the first five components. For PC1, high‐loading variables included: slope (0.938), canopy cover (0.858), shrub and herb cover (−0.681), slope position 1 (flat ground) (−0.537), slope position 3 (mid‐slope) (0.786), and vegetation type 2 (shrub‐grassland) (−0.718). This implies that Reeves' turtle avoids nesting in areas with steeper slopes, higher canopy cover, and mid‐slope positions, while favoring habitats with higher shrub/herb cover, lower slopes, and shrub‐grassland vegetation. Although PC2 showed no significant between‐group differences, variables such as distance from water source (0.935) and height above water source (0.949) exhibited high loadings. The negative mean difference (−0.161) in PC2 scores suggests a trend where turtles prefer nesting sites closer to water sources and at lower elevations relative to water. This aligns with results from paired samples *t*‐tests comparing selected and random quadrats. Other high‐loading factors in the remaining principal components, such as distance from human disturbance, leaf litter thickness, soil hardness, and shrub and herb height, showed no significant between‐group differences across the three analytical methods.

**TABLE 6:** Rotated factor matrix.

Variable	Factor 1	Factor 2	Factor 3	Factor 4	Factor 5
Slope	0.938	0.155	0.041	−0.063	0.166
Distance from human disturbance	−0.011	0.958	0.049	0.104	0.16
Canopy cover	0.858	0.214	0.05	0.177	−0.028
Distance from water source	0.158	0.935	−0.114	−0.151	−0.011
Height above water source	0.148	0.949	−0.141	−0.123	0.003
Soil hardness	−0.217	−0.266	0.618	−0.188	0.127
Leaf litter thickness	0.033	0.604	0.623	0.313	0.11
Shrub and herb height	0.167	0.095	−0.256	0.877	0.163
Shrub and herb cover	−0.681	−0.065	0.215	0.601	0.063
Slope position = 1	−0.537	−0.427	−0.553	0.219	−0.138
Slope position = 2	−0.158	−0.237	0.811	−0.181	−0.058
Slope position = 3	0.786	−0.037	−0.112	−0.144	0.256
Slope direction = 1	0.08	0.209	0.701	−0.15	−0.49
Slope direction = 2	−0.319	−0.33	−0.376	0.535	−0.427
Slope direction = 3	0.114	0.102	−0.097	0.045	0.934
Vegetation type = 1	−0.169	−0.277	−0.322	0.609	−0.448
Vegetation type = 2	−0.718	0.052	0.278	−0.153	0.444

### 3.2 Nest Characteristics, Clutch Size, and Egg Characteristics

During the field study, three nesting sites were predated upon before we discovered them, destroying the nest structures before they could be analyzed (nest predation rate of 42.86%), leaving only four intact nesting sites in the wild (Figure 1C). Statistical analysis was conducted on the four undamaged nest sites, revealing an average nest depth of the turtles of 89.87 ± 7.78 mm, average nest inner width of 69.02 ± 9.39 mm, average opening diameter of 52.46 ± 4.02 mm, average clutch size of 7.25 ± 0.48, average egg weight of 7.49 ± 0.29 g, average egg length of 35.63 ± 0.62 mm, and average egg width of 18.90 ± 0.28 mm. (Table 7).

**TABLE 7:** Nest characteristics, clutch size, egg characteristics, and laying dates at nine nest sites.

Turtle ID	Nest ID	Location	Success or failure	Egg‐laying date	Nest depth (mm)	Nest inner width (mm)	Opening diameter (mm)	Clutch size	Average egg weight (g)	Average egg length (mm)	Average egg width (mm)
1	1	Field	Failure	2022.5.26	111.80	66.26	55.36	8	8.00 ± 0.10	37.20 ± 0.47	19.26 ± 0.14
1	2	Field	Failure	2022.7.17	88.14	53.10	47.90	7	8.01 ± 0.07	37.44 ± 0.31	18.92 ± 0.10
2	3	Field	Failure	2022.6.11	84.22	96.00	62.34	6	4.67 ± 0.11	29.61 ± 0.68	16.32 ± 0.16
3	4	Field	Success	2022.7.12	75.32	60.70	44.24	8	8.65 ± 0.30	36.98 ± 0.40	20.50 ± 0.19
4	5	Field	Predation	2022.6.16	—	—	—	—	—	—	—
5	6	Field	Predation	2022.6.25	—	—	—	—	—	—	—
6	7	Field	Predation	2022.6.20	—	—	—	—	—	—	—
Mean ± SE					89.87 ± 7.78	69.02 ± 9.39	52.46 ± 4.02	7.25 ± 0.48	7.49 ± 0.29	35.63 ± 0.62	18.90 ± 0.28

### 3.3 Nesting Behavior

**FIGURE 2:**
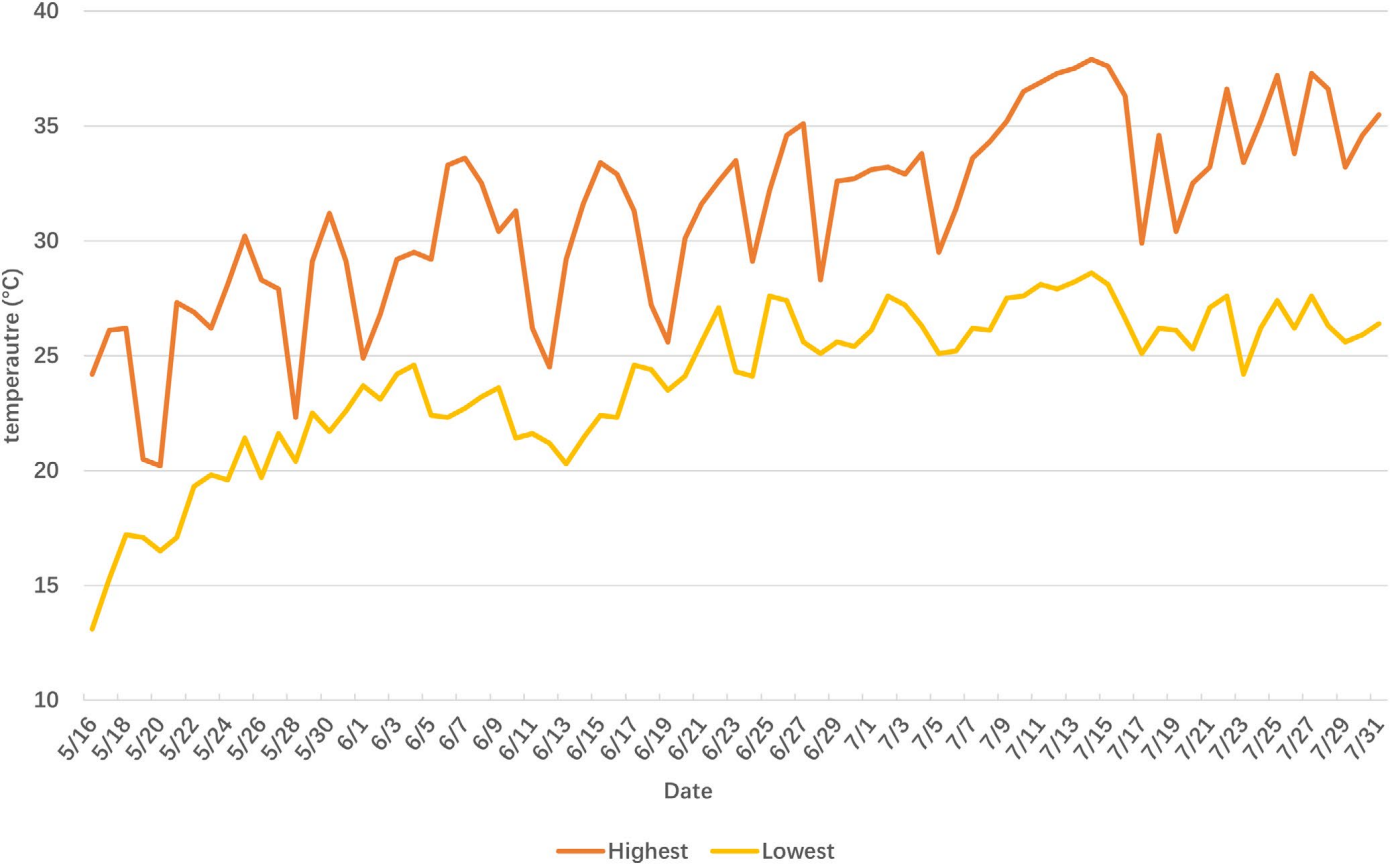
Temperature fluctuations within the enclosure during the breeding season.

**FIGURE 3:**
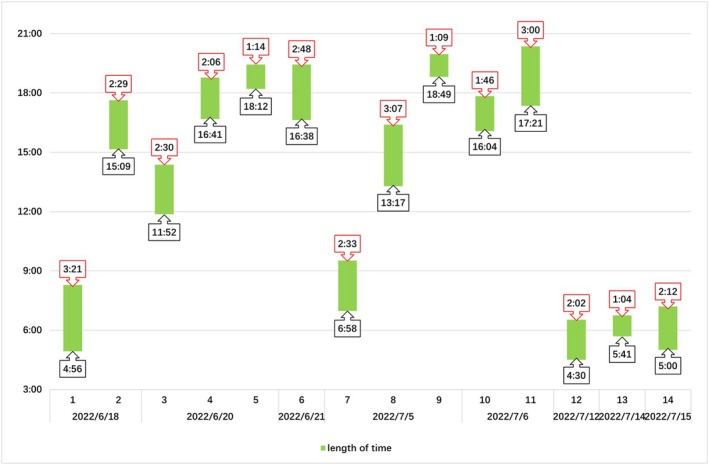
Nesting dates, length of time, and start time of 14 Reeves' turtles. The vertical axis represents time of day (03:00–21:00) and the horizontal axis denotes calendar dates during the breeding season. Horizontal green bars illustrate the temporal duration (the red labels above the green bars indicate the specific duration) of nesting behavior for individual turtles, with numerical labels below bars (black labels) indicating nest initiation times.

Observations were conducted within the enclosure to reveal the nesting behavior of 14 individuals. Combined with the field study results, the nesting season was determined to be from late May to late July, when temperatures were relatively high (Figure 2). The peak nesting period was from mid‐June to mid‐July. Nesting activity primarily occurred near dusk (15:00–21:00, *n* = 8) and dawn (03:00–9:00, *n* = 5), with only one female nesting at midday and no females nesting at night (21:00–03:00) (Figure 3). Some turtles nested on rainy days (*n* = 5), whereas others nested on sunny days (*n* = 9). Four of the turtles (#5, 9, 10, 13) did not nest successfully, and one of the turtles (#1) failed to nest on its first attempt but then successfully nested at a different location (Table 5). The nesting behavior was divided into five stages: landing (7.46 ± 1.57 min), excavation (78.20 ± 4.98 min), egg laying (18.20 ± 2.93 min), covering (43.60 ± 2.69 min), and return (2.30 ± 0.30 min) (Table 8).

**TABLE 8:** Duration of nesting behavior at each stage and weather of the day.

Numbers	Duration of each stage of behavior (min)					Weather
	LD	EX	EL	CO	RE	
1	6	7^a^	—	—	—	Rainy
1	16	62	32	32	3	Rainy
2	23	65	17	42	2	Rainy
3	2	83	14	49	3	Sunny
4	8	75	10	30	3	Sunny
5	2	72^a^	—	—	—	Sunny
6	14	83	30	39	2	Sunny
7	8	80	16	47	2	Rainy
8	2	100	31	53	1	Rainy
9	3	66^a^	—	—	—	Rainy
10	4	104^a^	—	—	—	Sunny
11	10	107	10	52	1	Sunny
12	4	63	14	39	2	Sunny
13	7	57^a^	—	—	—	Sunny
14	3	64	8	53	4	Sunny
Mean ± SE	7.46 ± 1.57	78.20 ± 4.98	18.20 ± 2.93	43.60 ± 2.69	2.30 ± 0.30	—

^a^ Indicates behavior that failed to proceed to the next stage.

#### 3.3.1 Landing

A female turtle planning to lay eggs comes to shore to search for suitable nesting sites. After crawling a distance, the turtle pauses to observe and sniff the surroundings for 10–20 s before continuing while occasionally sniffing the ground while moving. Upon finding a suitable site, the turtle uses its front legs to clear debris, creating a small cleared area. It then crawls forward and positions its hind legs above the cleared area to prepare for nest digging.

#### 3.3.2 Excavation

The turtle fixes its body with its front limbs, lifts the front part of its body, and angles its plastron at approximately 20° to the ground. The turtle gently digs 5–8 times with one hind limb, followed by a hard dig that clears the central soil on both sides. This set of actions requires approximately 30 s. It then repeats this set of actions with the other hind limb, alternating between them. As the nest deepens, the turtle switches to another digging method, inserting its left and right hind limbs alternately into the nest and removing the soil each time. To enable the hind limbs to reach the necessary depth, the turtle tilts its body towards the same side as the hind limb being used. Each side requires approximately 12 s. Once the nest reaches sufficient depth and size, the turtle can lay its eggs.

#### 3.3.3 Egg Laying

The turtle positions its hind limbs above the nest, with the cloaca hanging over the opening of the hole, maintaining the posture of nest‐digging, and begins to lay eggs. After laying one egg, its body trembles violently, and it extends one of its hind limbs to adjust the position of the egg. It takes approximately 15 s to lay each egg. Before preparing to lay the next egg, the turtle continues to dig the nest and expand it until the next egg reaches the cloaca.

#### 3.3.4 Covering

The turtle immediately begins to cover its nest, which is divided into three stages. In the first stage, it curls its hind limbs to gather nearby soil in the center of the nest. In the second stage, it stretches its hind limbs to collect soil from a greater distance. In the third stage, it leaves the nest and uses its hind limbs to cover the soil and debris in the center of the nest before using its feet to compact it.

#### 3.3.5 Return

The turtle walks with an unsteady gait, shakes its body, and returns to water without stopping.

## 4 Discussion

### 4.1 Selecting Nesting Sites With Low Canopy Cover May Increase the Average Nest Temperature but Carries Certain Risks

The results of the paired samples *t*‐test, discriminant analysis, and PCA all demonstrated that in the selection of nest sites, Reeves' turtles preferred locations with lower canopy cover. Canopy cover significantly influences the thermal environment of nest sites, exhibiting a negative correlation between canopy cover and nest site temperature (Sullivan et al. 2022). Similar to our study, painted turtles ( *Chrysemys picta* ) have demonstrated nesting behavior in areas with a lower average canopy cover than in other available habitats, resulting in higher average nest temperatures (Bodensteiner et al. 2023). This preference appears to offer both benefits and risks. The benefits include accelerating egg incubation, as a lower canopy increases the average temperature (Wang et al. 2010). In addition, as Reeve's turtle hatchlings overwinter in the nest (Fukada and Ishihara 1974), such a selection may increase the survival rate of hatchlings during overwintering, as winter nest temperatures are negatively correlated with summer vegetation cover. Indeed, nesting in densely vegetated environments has been associated with increased mortality of hatchlings overwintering in the nest (Weisrock and Janzen 1999). These results agree with those from a study on eastern box turtles ( *Terrapene carolina carolina* ) which found that hatchlings from nests with reduced canopy cover had higher survival rates during their first winter (Refsnider et al. 2021). The associated risks of this site selection include a female‐biased offspring sex ratio, as studies have shown that the thermal environment of nest sites influences the sex ratio in turtles that exhibit temperature‐dependent sex determination (TSD) (Vogt and Bull 1984; Janzen 1994). Reeves' turtles are a typical TSD species, with an average incubation temperature above 30°C leading to a female‐biased offspring sex ratio and temperatures below 26°C resulting in a male‐biased ratio (Du et al. 2007). In certain TSD species, extreme unisexuality can lead to population decline or extinction (Mitchell and Janzen 2010). However, evidence suggests that the situation might not be as severe as anticipated, with studies indicating that elevated temperatures and decreased hatchling survival in sea turtles result in a higher proportion of females, which increases the reproductive potential of future populations, positively influencing population growth (Schwanz et al. 2010; Santidrián Tomillo and Spotila 2020). Furthermore, although male turtles may become rarer, they tend to breed more frequently and actively seek mates from different sources, helping mitigate the effects of female‐biased hatchling sex ratios (Hays et al. 2022). Another risk is the increased risk of egg exposure to extreme high temperatures, as discussed in more detail in Section 4.4.

In summary, Reeve's turtles' preference for nesting in areas with lower canopy cover may be the result of a trade‐off. This choice likely maximizes fitness under constrained conditions by balancing the benefits and risks associated with such nesting behavior.

### 4.2 Choosing Nesting Sites Near Water Sources May Increase the Risk of Flooding but Offers Certain Benefits

The results of the paired samples *t*‐test indicated that distance from water sources and slope are key factors influencing nest site selection by Reeves' turtles. These two ecological factors exhibited high loadings in PC2 and PC1, respectively. To avoid the risk of nest flooding during nesting, the distance from the high‐tide line is a crucial factor in determining the nest site selection of green turtles ( *Chelonia mydas* ) (Patrício et al. 2018). Under these conditions, pig‐nosed turtles ( *Carettochelys insculpta* ) nest at high elevations (Doody et al. 2004). In addition, the slope determined the distance of the nest site from the water source. A gentler slope implies that female turtles must crawl a longer distance to reach the appropriate nesting height (Zare et al. 2012), whereas a steeper slope can result in the nest site being closer to the water source (Doody et al. 2004). However, in this study, whether the nest site was prone to flooding might not have been the primary factor influencing Reeves' turtle nest site selection. The turtles in this study showed a preference for nesting closer to water sources (15.19 ± 7.49 m) and on gentler slopes (4.63° ± 2.34°). According to the PCA results, these sites also exhibit lower slope positions. This behavior may indicate potential underlying benefits, such as minimizing the distance they must travel to the water, decreasing the chances of desiccation, and lowering the risk of predation (Zare et al. 2012; Martins et al. 2022). This site selection also reduces energy expenditure and predation risk faced by female turtles (Delaney et al. 2017). Studies have shown that predation risk influences the nest site selection of the Murray River turtle ( *Emydura macquarii* ). With increased predation risk, females tend to nest closer to the shoreline (Spencer and Thompson 2003). However, when predators are removed, females choose nesting sites that maximize offspring fitness (Spencer 2002). For Reeves' turtles, the choice to nest near water likely reflects a balance between maximizing offspring fitness and minimizing predation risk as although nesting closer to water sources increases the risk of nest flooding, the survival of the mother ensures increased future reproductive opportunities.

### 4.3 Temperature Influences the Occurrence of Nesting Behavior

During the nesting season, as temperatures increased, the nesting behavior of Reeves' turtles gradually increased in the study area, peaking from mid‐June to mid‐July. Reeves' turtles select seasons with higher average temperatures for nesting. Laying eggs under favorable thermal conditions and initiating hatching in warm environments reduces their incubation time (Du et al. 2006). The temperature also stimulates nesting behavior in freshwater turtles. For instance, for each 1°C increase in the average temperature, the nesting period of Blanding's turtles (*Emydoidea blandingii*) advances by 7 days, and nights when nesting behavior occurs have higher temperatures than typical nights (Buckardt et al. 2020).

Concerning nesting time, Reeves' turtles opted to nest in the afternoon, at dusk, or at dawn, with very few individuals nesting at noon. This bimodal nesting pattern has also been observed in other freshwater turtles, such as the Murray River turtle ( *Emydura macquarii* ), and may be attributed to avoidance of high temperatures (Bowen and Janzen 2005). In the study area, the average high temperature during the nesting season was 31.30°C ± 0.47°C, with extreme highs reaching 37.90°C. Typically, the temperature peaked between 11:00 and 13:00. Some turtle species such as the yellow‐spotted river turtle ( *Podocnemis unifilis* ) and Blanding's turtle (Escalona et al. 2019; Buckardt et al. 2020), choose to nest at night. However, Reeves' turtles did not exhibit nighttime nesting behavior (from 21:00 to 03:00), possibly due to the low nighttime temperature (23.96°C ± 0.38°C), which is unsuitable for nesting activities as it would require female turtles to expend more energy to remain active (Bulté and Blouin‐Demers 2010).

### 4.4 High Reproductive Failure Rate

Only one of seven wild nests hatched successfully, yielding a success rate of 14.3%. Three of the nests were predated, while the other three were found to have failed one month after incubation began (the soil around the failed nests was extremely dry and hard, with only eggshells remaining in the nests). Considering the prolonged high temperatures and low rainfall during the summer of 2022, the failure of these nests to hatch may be attributed to their exposure to extreme temperatures (Rafferty and Reina 2014; Turkozan et al. 2021). Extreme weather can cause abnormal nest temperatures; for instance, El Niño events reduce average precipitation and increase average temperatures, potentially affecting the nest temperatures of turtles in South America (Valenzuela 2021). Additionally, high temperatures and low rainfall can affect nest humidity. Owing to the high specific heat capacity of water, nests with higher humidity maintain more stable temperatures, which can increase hatching success to some extent (Parrott and David Logan 2010). The high reproductive failure rate of Reeves' turtles in the wild highlights their vulnerability to global warming, particularly the potential dual impact of hatching under extreme high temperatures and a female‐biased offspring sex ratio.

## 5 Conclusion

This study investigated the reproductive behavior and current reproductive status of Reeve's turtles in Qichun County, Hubei Province, China. The results showed that Reeves' turtles tend to choose nesting sites with gentle slopes, proximity to water sources, and low canopy cover. Such locations help shorten incubation time and reduce the risk of nest predation. Nesting behavior is primarily concentrated in June and July and mostly occurs in the early morning or late evening, possibly to avoid high temperatures. However, the reproductive success rate of Reeve's turtles in the study area is very low, mainly due to nest predation and extreme weather conditions. Future research should investigate the main nest predators, control their populations, and study the hatching process of Reeves' turtles to clarify the reasons for low hatching success rates. Moreover, due to the TSD characteristics of Reeves' turtles, it is important to consider how a female‐biased offspring sex ratio will affect their population. Long‐term monitoring of population dynamics is needed in the future to assess the extinction risk under the context of global warming. This study provides important information on the reproductive ecology of Reeve's turtles, which is important for understanding their adaptive capacity to environmental change and for the conservation of this species.

## Author Contributions

**Zihao Ye:** data curation (lead), investigation (lead), writing – original draft (lead), writing – review and editing (supporting). **Rongping Bu:** investigation (supporting), methodology (lead), supervision (lead), writing – original draft (supporting), writing – review and editing (supporting). **Hai‐Tao Shi:** funding acquisition (lead), methodology (lead), project administration (lead), resources (lead), supervision (supporting), writing – original draft (supporting), writing – review and editing (lead).

## Disclosure

Institutional Review Board statement: Fieldwork was carried out in strict accordance with the guidelines of the Animal Research Ethics Committee of the Hainan Provincial Education Centre for Ecology and Environment, Hainan Normal University (HNECEE 2021‐001), which conforms to the laws of China. None of the turtles were euthanized or injured in this study. All the turtles were released into the original capture area by the end of the study.

## Conflicts of Interest

The authors declare no conflicts of interest.

## Supporting information


Appendix S1

## Data Availability

The data that support the findings of this study are openly available in Dryad at: https://doi.org/10.5061/dryad.ttdz08m70.
